# Mesenchymal stem cells and pulmonary fibrosis: a bibliometric and visualization analysis of literature published between 2002 and 2021

**DOI:** 10.3389/fphar.2023.1136761

**Published:** 2023-07-04

**Authors:** Yanli Yang, Yu Chen, Yang Liu, Zongdi Ning, Zhaoliang Zhang, Yan Zhang, Ke Xu, Liyun Zhang

**Affiliations:** ^1^ Third Hospital of Shanxi Medical University, Shanxi Bethune Hospital, Shanxi Academy of Medical Sciences, Tongji Shanxi Hospital, Taiyuan, China; ^2^ Xinzhou People’s Hospital, Xinzhou, China

**Keywords:** bibliometrics, mesenchymal stem cells, pulmonary fibrosis, citespace, VOSviewers

## Abstract

**Introduction:** Pulmonary fibrosis (PF) is a severe disease that can lead to respiratory failure and even death. However, currently there is no effective treatment available for patients with PF. Mesenchymal stem cells (MSCs) have been recently shown to have therapeutic potential for PF. We analyzed the literature focused of MSCs and PF to provide a comprehensive understanding of the relationship between MSCs and PF.

**Methods:** We searched the Web of Science Core Collection database for literature from 2002 through 2021 that involved MSCs and PF. The included studies were then analyzed using CiteSpace and VOSviewers software.

**Results:** A total of 1,457 studies were included for analysis. Our findings demonstrated the following: 1) an increasing trend of MSC and PF research; 2) among the 54 countries/regions of author affiliations, the United States was the most frequent, and the University of Michigan (*n* = 64, 2.8%) was the top institution; 3) Rojas Mauricio published the most articles and *PLOS ONE* had the most related studies; and 4) keywords, such as idiopathic pulmonary fibrosis, mesenchymal stem cells, and systemic sclerosis, were listed more than 100 times, indicating the research trend. Other common keywords, such as inflammation, myofibroblasts, fibroblasts, aging, telomerase or telomere, and extracellular matrix demonstrate research interests in the corresponding mechanisms.1) The number of publications focused on MSCs and PF research increased during the study period; 2) Among the 54 countries/regions of author affiliations, most articles were published in the United States of America, and the University of Michigan (*n* = 64, 2.8%) had the largest number of publications; 3) Rojas Mauricio published the most articles and *PLOS ONE* had the most related studies; 4) Keywords, such as idiopathic pulmonary fibrosis, MSCs, and systemic sclerosis, were listed more than 100 times, representing a research trend. Other common keywords included inflammation, myofibroblasts, fibroblasts, aging, telomerase or telomere, and extracellular matrix.

**Discussion:** During the past 2 decades, MSCs have been proposed to play an important role in PF treatment. An increasing amount of literature focused on MSCs and PF research has been published. Our findings provide insight into the current status and research trends in the field of MSCs and PF research during the past 2 decades, which could help researchers understand necessary research directions. In the future, more preclinical and clinical studies should be conducted in this field to support the application of MSCs in the treatment of PF.

## Introduction

Pulmonary fibrosis (PF) is a major type of pulmonary disease that can lead to respiratory failure and death (Yang et al., 2021). PF is characterized by fibroblast proliferation and extracellular matrix (ECM) aggregation, resulting in structural abnormality during aberrant repair of normal alveolar tissue injury. The clinical manifestations of PF include hypoxemia, dyspnea, respiratory failure, and even death. The etiology of PF is not specific, but there are many known contributing factors, such as autoimmune disease, viral infection, silica inhalation, long-term smoking, radiation, chemotherapy, and genetic mutations.

During the past 2 decades, significant progress has been made in understanding the pathogenesis of PF, especially idiopathic pulmonary fibrosis (IPF). IPF is the most common form of PF, and is defined as a progressive lower respiratory disease that usually affects adults over the age of 40. Alveolar epithelial dysfunction is considered a critical step in the initiation of IPF. Given that genetic factors can influence the integrity of epithelial cells, and environmental and aging-related factors can trigger epigenetic reprogramming, epithelial cells are likely to be damaged and abnormally activated by these factors (Winters et al., 2019). Activated epithelial cells secrete a large number of cytokines, such as tumor growth factor-beta (TGF-β), to promote fibroblast migration and proliferation, as well as stimulate fibroblast differentiation into myofibroblasts, which then secrete a large amount of ECM, resulting in ECM deposition (Mei et al., 2021). IPF has a poor prognosis, with an average life expectancy of 3–5 years after diagnosis without treatment (Lederer and Martinez, 2018; Raghu et al., 2018) and mortality exceeding that of many malignant tumors (Karampitsakos et al., 2017). Regardless of improvements in drug therapy, such as corticosteroids, endothelin antagonists, and pirfenidone, there is still no highly effective treatment for PF. Lung transplantation can improve the quality of life and survival of patients, but various challenges exist with this treatment, including shortage of donor organs, immune rejection, and surgical complications (Richeldi et al., 2017; Shenderov et al., 2021). Thus, relieving symptoms and delaying disease progression remain the primary management strategies (Li et al., 2021). Therefore, there is an urgent need to develop new therapeutic strategies that are safe and effective for PF treatment.

Mesenchymal stem cells (MSCs) are multipotent stem cells with immunomodulatory effects and paracrine functions. MCSs can promote tissue regeneration by secreting a variety of cytokines and soluble factors with anti-inflammatory and antifibrosis properties. MSCs not only reduce the levels of inflammatory factors and the deposition of activated fibroblasts and collagen, but they can also inhibit epithelial-mesenchymal transition (EMT) and promote epithelial repair, suggesting that MSCs may be a novel therapeutic target for PF (Chambers et al., 2014; Averyanov et al., 2020; Zhao et al., 2021). As such, advances have been made in utilizing MSCs to promote repair and regeneration of lung tissue.

Bibliometrics is a type of literature analysis that allows for the quantitative and qualitative review of scientific output within a specific research field (Wang et al., 2019; Ke et al., 2020). More specifically, bibliometrics utilizes characteristics of the literature, such as authors, keywords, journals, countries, institutions, and references related to the target research area (Ke et al., 2020), that can be analyzed and visualized using informatics tools, such as CiteSpace (Synnestvedt et al., 2005) and VoSviewer (Ke et al., 2020; Yeung et al., 2020; Teles et al., 2021; Wu et al., 2021). To the best of our knowledge, a bibliometric analysis of the literature focused on the application of MSCs to treat PF has not been performed (Zhang et al., 2020; Wu et al., 2021). Due to the urgency of developing effective therapies for PF and the potential of targeting MSCs for PF treatment, we conducted a comprehensive analysis of the scientific literature on MSCs in the treatment of PF during the past 2 decades. Our analysis provides important insights into the state of the current literature and directions for future research on MSCs and PF.

## Materials and methods

### Search strategy

**FIGURE 1:**
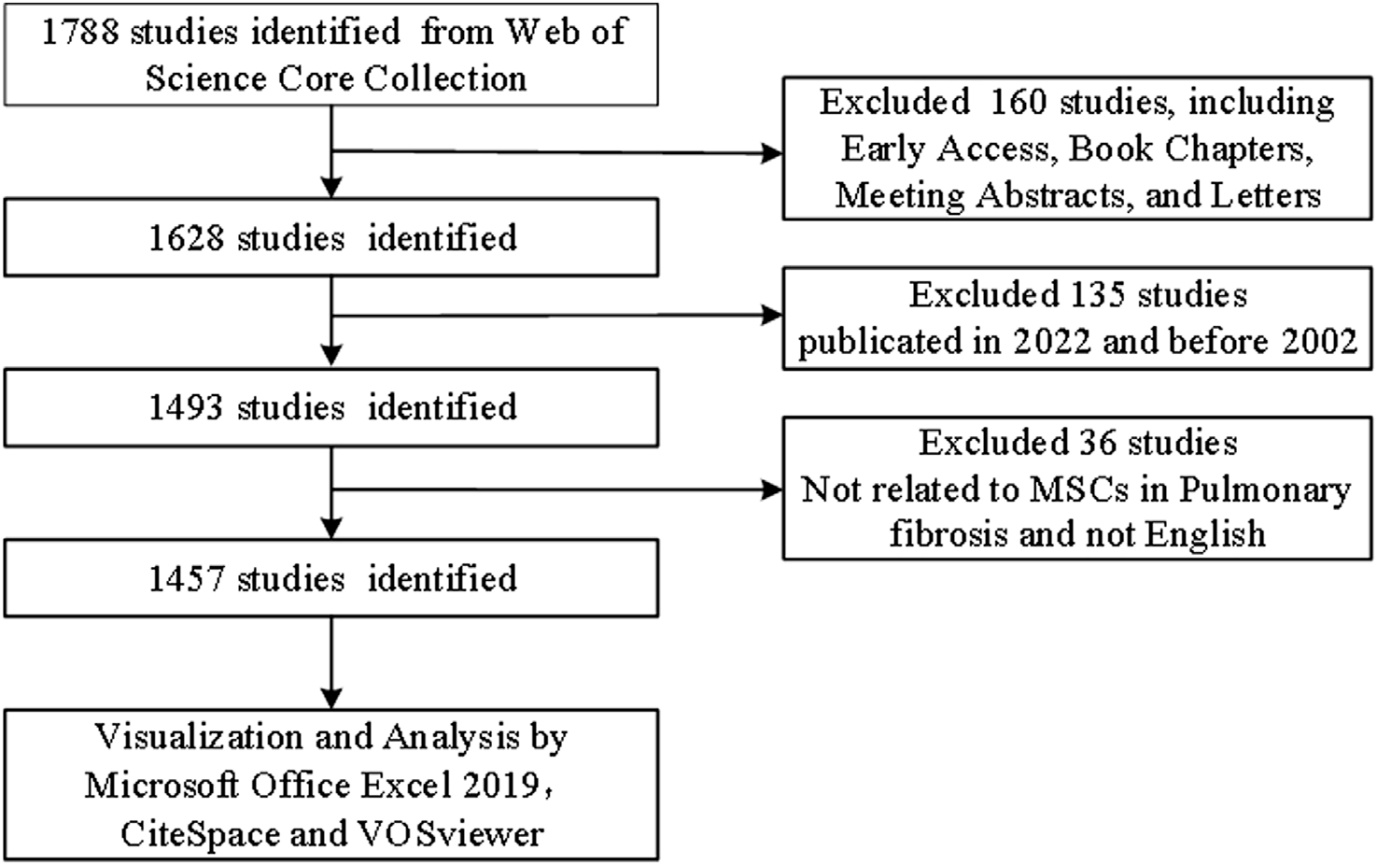
Flowchart of literature selection.

We conducted a search of the Web of Science Core Collection (WoSCC, https://www.webofscience.com/wos/woscc/basic-search) using the following terms: “TS = [(“Stem Cell*"OR “Stromal Cell*") AND TS = (“ILD” OR “interstitial lung disease” OR “pulmonary Fibrosis”)]” on 5 August 2022. Only articles and reviews published in English were selected. Other document types, such as early access, book chapters, meeting abstracts, and letters, were excluded. The details of literature selection are provided in Figure 1.

### Data analysis

VOSviewer (version 1.6.18) was used to analyze the cooperation between countries and institutions, journals and co-cited journals, authors and co-cited authors, keyword co-occurrence, and network visualization map. In the map, nodes represent various items, such as country, institution, journal, and author, with node size representing the number of included items. The thickness of the lines linking different nodes reflects the degree of cooperation or co-citation of projects (Zhang et al., 2020; Wu et al., 2021).

CiteSpace (version 6.1. R1) was employed to draw the dual-map overlay of scientific journals that published articles on MSCs research in the field of PF. CiteSpace was also used to analyze references with citation bursts. Microsoft Office Excel 2019 was used to manage the data and assist the analysis.

## Results

### Quantity of literature

**FIGURE 2:**
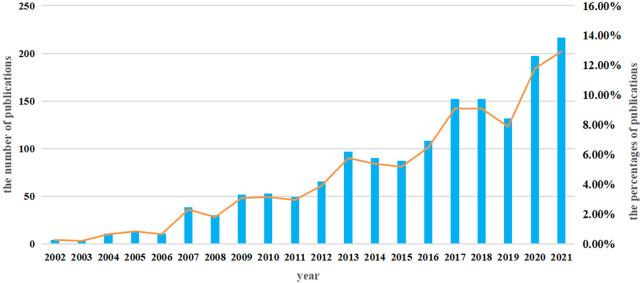
Quantity of literature between 2002 and 2021.

A total of 1,457 studies (984 articles and 473 reviews) that included MSCs and PF as keywords were identified from 2002 to 2021. The number of studies increased steadily from 2002 to 2015, with a more rapid increase beginning in 2016; over 100 studies were published yearly (Figure 2).

### Institution and country

**FIGURE 3:**
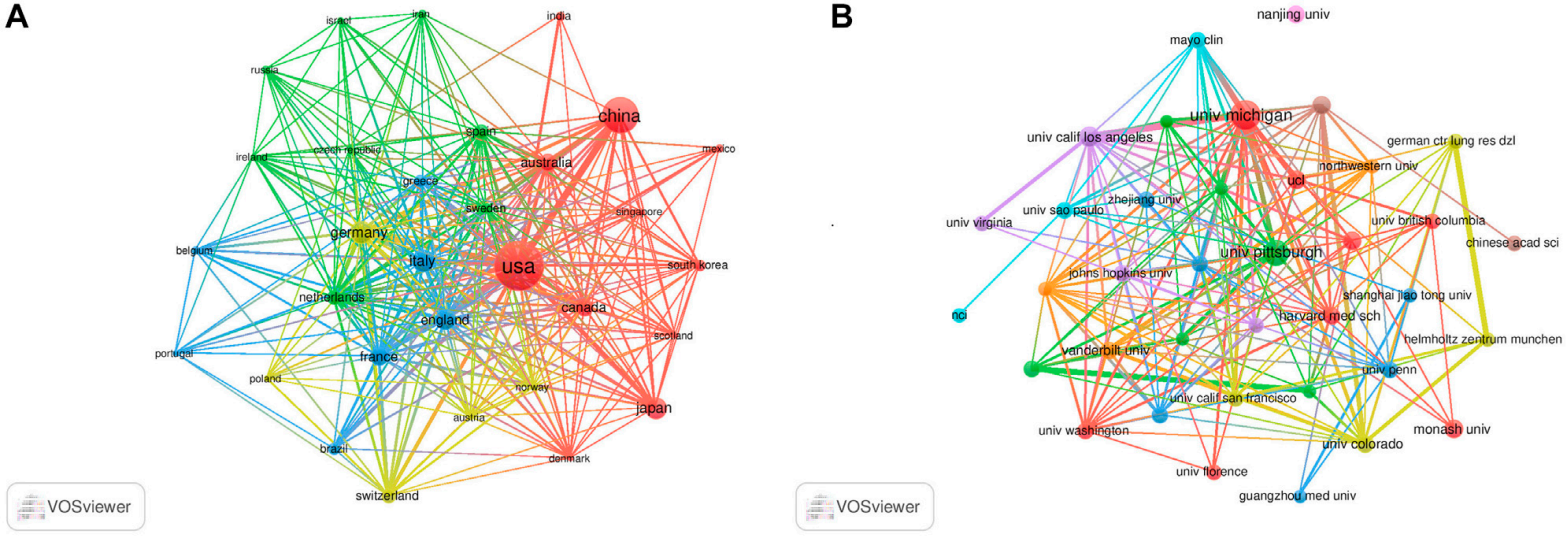
Visualization results of countries **(A)** and institutions **(B)** in the field of MSCs and PF research.

The authors were distributed among 54 countries and 1,661 institutions. As shown in Table 1, the top country was the United States of America (United States) (*n* = 632, 30.5%), followed by China (*n* = 294, 14.2%), Germany (*n* = 124, 6.0%), Italy (*n* = 118, 5.7%), and Japan (*n* = 105, 5.1%). Literature from the United States and China accounted for almost half of all the retrieved studies. Countries with frequencies ≥6 (*n* = 31) were selected for visualization analysis, and a cooperation network was constructed according to the number and relationship of publications in each country/region (Figure 3A). Positive cooperation was found between China and the United States, as well as Australia and Japan. The United States also had close collaboration with Japan, Germany, France, and Italy**.**

**TABLE 1:** Top 10 countries and institutions in the field of MSCs and PF research.

Rank	Country	Counts	Institution	Counts
1	United States (North America)	632 (30.5%)	University of Michigan (United States)	64 (2.8%)
2	China (Asia)	294 (14.2%)	University of Pittsburgh (United States)	47 (2.0%)
3	Germany (Europe)	124 (6.0%)	University of California Los Angeles (United States)	29 (1.3%)
4	Italy (Europe)	118 (5.7%)	Monash University (Australia)	25 (1.1%)
5	Japan (Asia)	105 (5.1%)	Harvard University (United States)	24 (1.0%)
6	The United Kingdom (Europe)	95 (4.6%)	University of Toronto (Canada)	23 (1.0%)
7	France (Europe)	79 (3.8%)	University of Colorado System (United States)	21 (0.9%)
8	Canada (North America)	70 (3.4%)	Vanderbilt University (United States)	21 (0.9%)
9	Australia (Oceania)	68 (3.3%)	Nanjing University (China)	21 (0.9%)
10	Netherlands (Europe)	47 (2.3%)	University of Sao Paulo (Canada)	20 (0.9%)

The top 10 institutions publishing on MSCs and PF were from four countries. The majority of these institutions (6/10) was in the United States. The University of Michigan (*n* = 64, 2.8%), University of Pittsburgh (*n* = 47, 2.0%), University of California Los Angeles (*n* = 29, 1.3%), Monash University (*n* = 25, 1.1%), and Harvard University (*n* = 24, 1.0%) were the top five institutions. Institutions with frequencies ≥13 (*n* = 37) were selected for visualization analysis, and a cooperation network was constructed according to the number and relationship of each institution (Figure 3B). According to the results, there was a close association between the University of Michigan and the University of California Los Angeles, and similar findings were observed between German CTR Lung Res DZL and Helmholtz Zentrum Munchen, and the University of Michigan and Mayo Clinic. Although a large number of studies was published from Nanjing University, the collaboration of Nanjing University with other institutions was modest.

### Journals and co-cited journals

**FIGURE 4:**
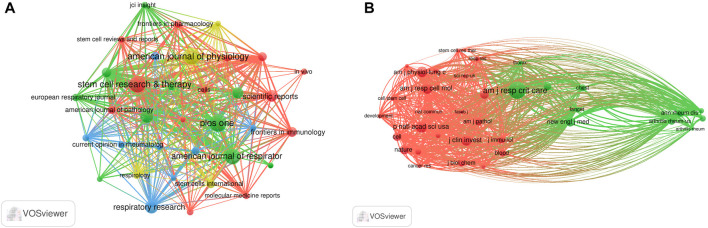
Visualization results of journal **(A)** and co-cited journal **(B)** in the field of MSCs and PF research.

The literature were published in 508 academic journals. Most studies (*n* = 39, 7.7%) were published in *PLOS ONE*, followed by the *American Journal of Physiology—Lung Cellular and Molecular Physiology* (*n* = 38, 7.5%), *Stem Cell Research and Therapy* (*n* = 37, 7.3%), and the *American Journal of Respiratory Cell and Molecular Biology* (*n* = 34, 6.7%). Among the top 15 journals, the *American Journal of Respiratory and Critical Care Medicine* had the highest impact factor (IF = 30.528), followed by the *Journal of Clinical Investigation* (IF = 19.456). Journals with ≥8 publications were used to construct the journal network map (Figure 4A).

Among the top 15 co-cited journals, five journals had a total citation of more than 2,000 times (Table 2). The *American Journal of Respiratory and Critical Care Medicine* (*n* = 4,307) was the most frequently cited journal, followed by *Proceedings of the National Academy of Sciences* (*n* = 2,624), the *American Journal of Respiratory Cell and Molecular Biology* (*n* = 2,411), and the *Journal of Clinical Investigation* (*n* = 2,399). Among the top 15 journals, the *New England Journal of Medicine* had the highest impact factor (IF = 176.079), followed by *Nature* (IF = 69.504). Journals with ≥500 co-citations were analyzed to determine the co-citation network (Figure 4B). In summary, a positive co-citation relationship was observed between the *American Journal of Respiratory and Critical Care Medicine* and *Chest*, as well as *Proceedings of the National Academy of Sciences* and the *New England Journal of Medicine*.

**TABLE 2:** Top 15 journals and co-cited journals in the field of MSCs and PF research.

Rank	Journal	Count	IF	Q	Co-cited journal	Co-citation	IF	Q
1	*PLOS ONE*	39 (7.7%)	3.752	2	*American Journal of Respiratory and Critical Care Medicine*	4307	30.528	1
2	*American Journal of Physiology-Lung Cellular and Molecular Physiology*	38 (7.5%)	4.406	2	*Proceedings of the National Academy of Sciences*	2624	12.779	1
3	*Stem Cell Research and Therapy*	37 (7.3%)	8.079	1	*American Journal of Respiratory Cell and Molecular Biology*	2411	7.448	1
4	*American Journal of Respiratory Cell and Molecular Biology*	34 (6.7%)	7.748	1	*Journal of Clinical Investigation*	2399	19.456	1
5	*American Journal of Respiratory and Critical Care Medicine*	26 (5.1%)	30.528	1	*American Journal of Physiology-Lung Cellular and Molecular Physiology*	2234	6.011	1
6	*Respiratory Research*	25 (5.0%)	7.162	1	*Nature*	2001	69.504	1
7	*Scientific Reports*	22 (4.3%)	4.996	1	*PLOS ONE*	1922	2.74	1
8	*International Journal of Molecular Sciences*	21 (4.1%)	6.208	1	*The New England Journal of Medicine*	1824	176.079	1
9	*Journal of Clinical Investigation*	17 (3.3%)	19.456	1	*American Journal of Pathology*	1,675	5.770	1
10	*Frontiers in Immunology*	17 (3.3%)	8.786	1	*Cell*	1,644	66.850	1
11	*Stem Cells Translational Medicine*	15 (3.0%)	7.655	1	*European Respiratory Journal*	1,564	33.795	1
12	*Stem Cells International*	14 (2.8%)	5.131	2	*Annals of the Rheumatic Diseases*	1,544	27.973	1
13	*American Journal of Pathology*	13 (2.6%)	5.770	1	*Journal of Immunology*	1,527	5.426	2
14	*Rheumatology*	13 (2.6%)	7.046	1	*Journal of Biological Chemistry*	1,517	5.486	2
15	*Clinical and Experimental Rheumatology*	13 (2.6%)	4.862	2	*Blood*	1,449	25.476	1

**FIGURE 5:**
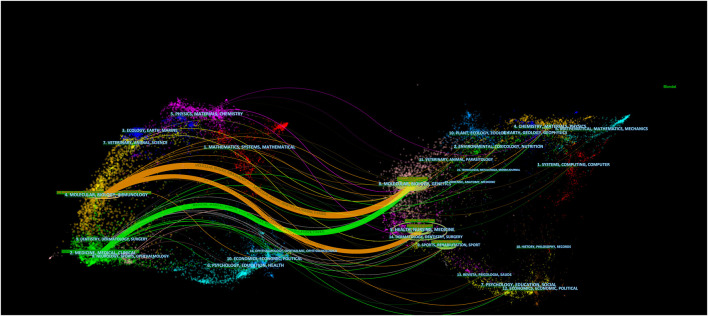
Dual-map overlay of journals related to the field of MSCs and PF research.

The dual-map overlay of journals was used to analyze the relationship of citations between journals and co-cited journals (citing journals, left; cited journals, right) (Chen, 2017). The orange and green lines are the main application paths. The orange lines indicate studies published in molecular, biology, genetics, health, nursing, and medicine journals; the primary citations were in molecular, biology, and immunology journals. The green lines indicate studies published in molecular, biology, and genetics journals; the primary cited studies were in medicine, medical, and clinical journals (Figure 5).

### Authors and co-cited authors

**FIGURE 6:**
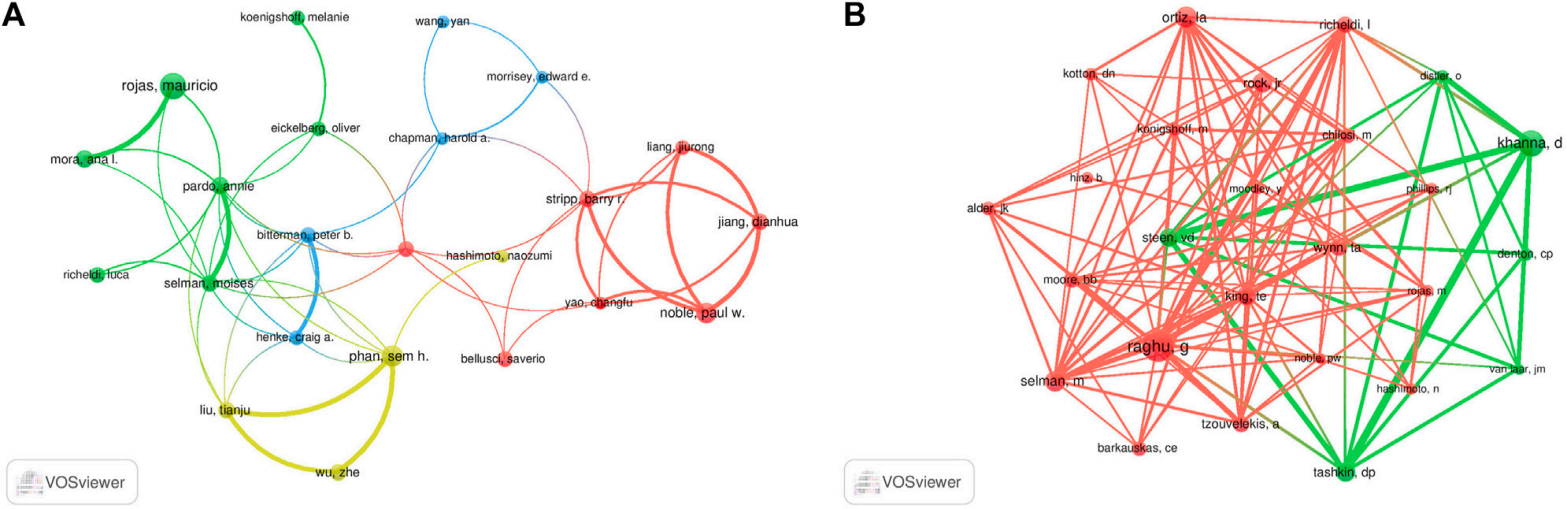
Visualization results of author **(A)** and co-cited author **(B)** in the field of MSCs and PF research.

A total of 7,842 authors were retrieved during the literature search. As shown in Table 3, the first two authors have published at least 15 studies. A collaboration network was conducted with the authors who published ≥7 papers. According to the number of relevant published studies, Rojas Mauricio, Xiaodong Han, Phan Sem H, and Weiss Daniel J had the largest nodes, and a close collaboration was observed between Rojas Mauricio and Mora Ana L, Noble Paul W and Stripp Barry R, and Henke Craig A and Bitterman Peter B (Figure 6A).

**TABLE 3:** Top 10 authors and co-cited authors in the field of MSCs and PF research.

Rank	Authors	Count	Co-cited authors	Citations
1	Rojas Mauricio	19	Raghu G	494
2	Xiaodong Han	15	Khanna D	373
3	Phan Sem H	14	Ortiz LA	296
4	Weiss Daniel J	14	Selman M	269
5	Blasco Maria A	13	King TE	266
6	Noble Paul W	13	Rock JR	258
7	Epperly Michael W	12	Tashkin DP	254
8	Greenberger Joel S	12	Steen VD	239
9	Hong Wang	12	Wynn TA	222
10	Strieter Robert M	12	Richeldi L	209

Co-cited authors are defined as authors who are co-cited in a series of publications. Among the 43,654 co-cited authors, 11 of them were co-cited >200 times. Raghu G had the highest number of co-citations (*n* = 494), followed by Khanna D (*n* = 373) and Ortiz La (*n* = 296). Authors with ≥120 co-citations were analyzed using the co-citation network graph (Figure 6B). Many positive cooperative relationships among different co-cited authors were found, such as Khanna D and Steen VD, and Tashkin Dp and Raghu G and King Te.

### Co-cited references

**FIGURE 7:**
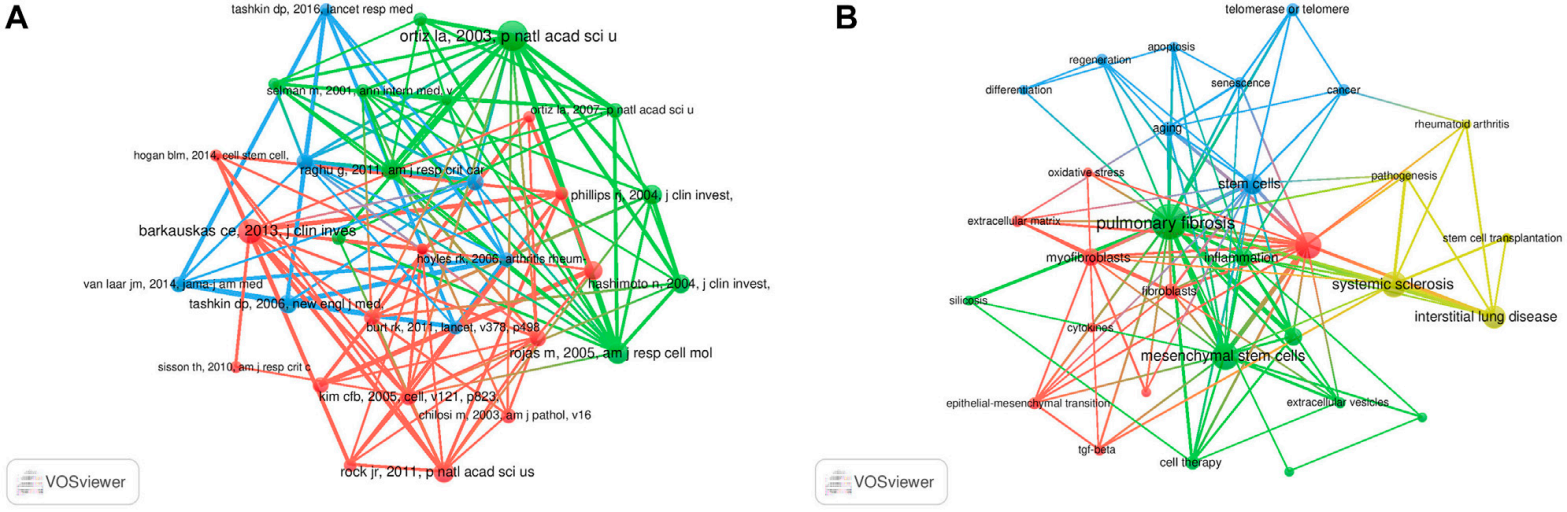
Visualization results of co-cited references **(A)** and Cluster analysis of keywords **(B)** in the field of MSCs and PF research.

Co-cited references are defined as those references cited together by other publications. A total of 64,747 co-cited references were found in the publications related to MSCs and PF research. Among the top nine co-cited references related to MSCs and PF research (Table 4), each reference was co-cited ≥100 times. References with ≥60 co-citations were analyzed using the co-citation network diagram (Figure 7A). The data showed that “Rojas M, 2005, Am J Resp Cell Mol” had active co-citation relationships with “Ortiz La, 2003, P Natl Acad Sci United States”.

**TABLE 4:** Top 10 co-cited references in the field of MSCs and PF research.

Rank	Co-cited references	Citations
1	Ortiz La, 2003, P Natl Acad Sci United States, v100, p8407	202
2	Barkauskas Ce, 2013, J Clin Invest, v123, p3025	153
3	Rojas M, 2005, Am J Resp Cell Mol, v33, p145	131
4	Rock Jr, 2011, P Natl Acad Sci United States, v108, pe1475	122
5	Phillips Rj, 2004, J Clin Invest, v114, p438	120
6	Raghu G, 2011, Am J Resp Crit Care, v183, p788	118
7	Hashimoto N, 2004, J Clin Invest, v113, p243	109
8	Tashkin Dp, 2006, New Engl J Med, v354, p2655	109
9	Armanios My, 2007, New Engl J Med, v356, p1317	108
10	Richeldi L, 2014, New Engl J Med, v370, p2071	97

### References with citation bursts

**FIGURE 8:**
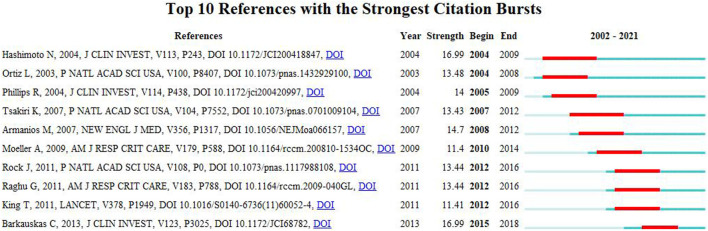
Top 10 references with strong citation bursts. Red bar indicates high citations in that year.

A citation burst refers to references that are frequently cited by scholars in a particular field over a period of time. Ten references with strong citation bursts were detected using CiteSpace, and the minimum burst duration of literature related to MSCs and PF research was 3 years. In Figure 8, the red bar represents the reference burst. The earliest citation burst occurred in 2004, and the latest occurred in 2015. Among these references, “Bone marriage-derived progenitor cells in Pulmonary Fibrosis” (authored by Hashimoto N et al.) had the strongest citation burst (strength = 16.99), which appeared between 2004 and 2009. The second reference was “Mesenchymal stem cell engraftment in lung is enhanced in response to bleomycin exposure and ameliorates its Fibrotic Effects” (authored by Ortiz L et al.; strength = 13.48; from 2004 to 2008). Overall, the burst strength of the top 10 references ranged from 11.4 to 16.99, while duration was from 3 to 5 years. Table 5 summarizes the main research content of the 10 references with the strongest citation bursts from Figure 8.

**TABLE 5:** The top 10 references with strong citations bursts.

Rank	Main content	Strength
1	The collagen-producing lung fibroblasts in pulmonary fibrosis can also be derived from bone marrow progenitor cells	16.99
2	Murine MSCs home to lung in response to injury, adopt an epithelium-like phenotype, and reduce inflammation and collagen deposition in lung tissue of mice challenged with Bleomycin	13.48
3	Murine CD45+Col I + CXCR4+ fibrocytes also traffic to the lungs in response to a bleomycin challenge. Maximal intrapulmonary recruitment of CD45+Col I + CXCR4+ fibrocytes directly correlated with increased collagen deposition in the lungs	14
4	Mutations in TERT or TERC that result in telomere shortening over time confer a dramatic increase in susceptibility to adult-onset idiopathic pulmonary fibrosis	13.43
5	Mutations in the genes encoding telomerase components can appear as familial idiopathic pulmonary fibrosis	14.7
6	Circulating fibrocytes are progenitors for fibroblasts and are thought to participate in the pathogenesis of lung fibrosis	11.4
7	They argue against a major direct contribution of epithelial cells to fibroblast foci through the process of epithelial–mesenchymal transition. Rather, they support models in which resident stromal cells, of commonly underestimated heterogeneity, expand under fibrogenic conditions	13.44
8	Mesenchymal stem cells migrate to the lung, adopt an epithelium-like phenotype, and reduce fibrosis in bleomycin-injured lungs from mice	13.44
9	The therapeutic potential of AEC type II derived from human embryonic stem cells was studied in the mouse model of bleomycin-induced injury. These cells differentiated into type I pneumocytes, and abrogated the inflammatory and fibrotic response	11.41
10	SFTPC + AEC2s, as a population, function as alveolar progenitors and long-term stem cells in the adult lung	16.99

### Hotspots and Frontier research areas

The high-frequency keywords in the field of MSCs and PF research are shown in Table 6, amongst which IPF, MSCs, and systemic sclerosis appeared >100 times, representing future research directions. Other keywords, such as inflammation, myofibroblasts, fibroblasts, aging, telomerase or telomere, extracellular matrix, and epithelial-mesenchymal transition, appeared >20 times, which may point to areas of mechanistic studies involving MSCs and PF.

**TABLE 6:** Top 20 keywords on research of MSCs in PF.

Rank	Keywords	Counts	Rank	Keywords	Counts
1	Pulmonary fibrosis	198	11	Aging	30
2	Idiopathic pulmonary fibrosis	110	12	Cell therapy	28
3	Mesenchymal stem cells	104	13	Telomerase or telomere	24
4	Systemic sclerosis	102	14	Extracellular matrix	22
5	Interstitial lung disease	80	15	Regeneration	22
6	Stem cells	66	16	Epithelial-mesenchymal transition	21
7	Inflammation	49	17	Senescence	19
8	Myofibroblasts	47	18	Extracellular vesicles	18
9	Bleomycin	45	19	TGF-β	18
10	Fibroblasts	37	20	Pathogenesis	16

The cluster analysis of keywords was performed using VOSviewer software. Each circle represents a keyword, the size of the circle indicates the extent of positive correlation with the frequency of the keyword, and the line thickness linking the circles indicates the degree of correlation between these keywords. A total of four clusters were obtained (Figure 7B), suggesting four main research directions. The yellow cluster includes systemic sclerosis, rheumatoid arthritis, interstitial lung disease, pathogenesis, and stem cell transplantation. The blue cluster includes aging, stem cells, telomerase or telomere, regeneration, apoptosis, and cancer. The red cluster includes myofibroblasts, fibroblasts, EMT, tumor TGF-β, and cytokines. The green cluster includes PF, MSCs, inflammation, and extracellular vesicles (EVs).

The current status of the research Frontier and the challenges of MSCs and PF are discussed in detail. Since the first isolation by Friedenstein et al. (1970) in 1970, bone marrow-derived mesenchymal stem cells (BM-MSCs) have become the most common source of multipotent cells for transplantation in preclinical and clinical trials. However, harvesting BM-MSCs is a painful, invasive process and carries a risk of viral exposure. In addition, the number, differentiation potential, and maximum longevity of BM-MSCs decreases with age (Bustos et al., 2014; Cárdenes et al., 2018; Nehlin et al., 2019). Compared to BM-MSCs, umbilical cord derived mesenchymal stem cells (UC-MSCs) have a similar profile, are easier to harvest, have no ethical or safety concerns, and are more rapidly refreshed (Ding et al., 2015). At present, many preclinical studies have confirmed that MSCs are safe and effective in the treatment of PF. One meta-analysis (Li et al., 2021) of 24 preclinical studies involving MSCs-targeted PF treatment showed that MSC treatment significantly improved patient survival rate and alleviated PF in animal models. All published clinical trials that have applied MSCs for PF treatment have confirmed the safety of MSC treatment and the benefits for some patients (Table 7). Of the clinical studies included in Table 7, all were collected from clinicalTrials.gov, except the fifth study (No. 5). Recently, some progress has been made in engineering MSCs to regulate and guide their behavior. For example, endocytic superparamagnetic iron oxide nanoparticles have been shown to improve MSC targeting to the lung (Silva et al., 2018).

**TABLE 7:** Summary of current clinical trails in MSCs and MSC-EVs for PF.

NO	Trail ID	Disease	Title	Inclusion criteria	No. Of patients	Cell type	Route		No of cells per infusion	No. of infusions	Safety outcome	Efficacy outcome	Phase	Locations
1	NCT02594839	IPF	First-in-human high-cumulative-dose stem cell therapy in idiopathic pulmonary fibrosis with rapid lung function	Progressive IPF (FVC or DLco decline of≥10% dur- ing the last year, current FVC≥40%, current DLco≥20%)	20	Allogeneic	IV		Group 1: 10 patients, 1.6×10^9^ cells	Multiple infusions of 200×10^6^ cells over 39 weeks	The main adverse reactions were mild fever and chills, and did not require study discontinuation.	FVC decline was significantly improved in the MSCs treatment group at 52 weeks (from −13.8% to +3.7%), as compared with the placebo group (from −11.7% to −9.5%). But, in the MSCs therapy group	I/IIa	Russian Federation
			decline (Averyanov et al., 2020)			BM-MSCs			Group 2: 10 patients, placebo			4 patiengts had a continuing decline of FVC and DLco >10% over the observation period.		
2	NCT01919827	IPF	Endobronchial autologous bone marrow– mesenchymal stromal cells in idiopathic	mild-to-moderate IPF (FVC⩾50% predicted and DLco⩾35% pred)	13	Autologous BM-MSCs	Endobr-onchial infusion		(10–100)×	Multiple infusions	No treatment-related severe adverse events were observed during follow-up.	Compared to baseline, the mean forced vital capacity showed an initial decline of 8.1% at 3 months. The number of patients without functional progression was six (46%) at 3 months and three (23%) at 12 months.	I	Spain
			pulmonary fibrosis: a phase I trial (Campo et al., 2021)						10^6^ cells					
3	NCT01385644	IPF	A phase 1b study of placenta-derived mesenchymal stromal cells in patients with idiopathic pulmonary fibrosis (Chambers et al., 2014)	Moderate to severe disease (FVC≥50%	8	Allogene placenta-derived MSCs	IV		Group 1:4 patients,1×10^6^ cells	Single-dose	Most adverse events were mild and self-limiting.	At 6 months FVC, DLco, 6 MWD and CT fibrosis score were unchanged compared with baseline.	Ib	Australia
				DLco≥25%					Group 2:4 patients,2×10^6^ cells					
				Honeycomb-ing>5%										
				in≤3/6 lung										
				zones										
4	NCT02013700	IPF	Allogeneic Human Mesenchymal Stem Cells in Patients With Idiopathic Pulmonary	Mild to moderate disease (FVC≥50%	9	Allogeneic	IV		Group 1:3 patients,2×10^7^ cells	Single-dose	There were no instances of treatment-emergent adverse events.	At 60 weeks after MSCs injection, there was a 3.0% mean decline in % predicted FVC and 5.4% mean decline in % predicted DLco.	I	United States
			Fibrosis via Intravenous Delivery (AETHER): A Phase I Safety Clinical Trial (Glassberg et al., 2017)	DLco≥30%)		BM-MSCs			Group 2:3 patients, 10^8^cells					
									Group 3:3 patients,2×10^8^ cells					
5	unknown	IPF	A prospective, non-randomized, non-placebo-controlled	Mild to moderate disease (FVC>50%,DLco>35%)	14	Autologous AD-MSCs	endobr-onchial infusions		5×10^5^ cells/kg	3 infusions 1 month apart	Minor adverse events, mostly related to Bronchoscopy.	No fibrosis exacerbation	Ib	Greece
			phase Ib clinical trial											
			to study the safety											
			of adipose derived											
			stromal cells-stromal											
			vascular fraction in idiopathic pulmonary											
			fbrosis (Tzouvelekis et al., 2013)											
6	NCT05468502	IPF	Phase I/IIa Clinical Trial of Human Umbilical Cord Mesenchymal Stem Cell Injection in the Treatment of Idiopathic Pulmonary Fibrosis (IPF)	Mild to moderate disease ((FVC>50%,DLco>35%)	18	Allogeneic hUC-MSCs	endobr-onchial infusions		Group 1:3 patients6.0*10^6^ cells	Single-dose	No results posted	No results posted	I/IIa	China
									Group 2:3 patients, 3.0*10^7^ cells					
									Group 3:3 patients,6.0×10^7^ cells					
									Group 4:3 patients9.0*10^6^ cells					
7	NCT05016817	IPF	Safety of Cultured Allogeneic Adult Umbilical Cord Derived Mesenchymal Stem Cell Intravenous Infusion for IPF	Diagnosis of Idiopathic Pulmonary Fibrosis	20	Allogeneic hUC-MSCs	IV		10^8^ cells	Single-dose	No results posted	No results posted	I	Antigua and Barbuda
														Argentina
														Mexico
8	NCT03929120	ILD	Allogeneic Bone Marrow Mesenchymal Stem Cells for Patients With Interstitial Lung Disease (ILD) and Connective Tissue Disorders (CTD)	Patients with new diagnosis of ILD associated with CTD, ANCA associated vasculitis or IPAF or established diagnosis of ILD associated with CTD under conventional therapy for at least 6 months but less than 24 months, with no evidence of improvement.	10	Allogeneic BM-MSCs	IV		(0.5–1)×10^6^ cells/kg	Single-dose	No results posted	No results posted	I	United States

IPF, idiopathic pulmonary fibrosis; FVC, functional vital capacity; DLco, diffusing capacity of the lung for carbon monoxide; BM-MSCs, bone marrow-derived mesenchymal stem cells; IV, intravenous; AD-MSCs, adipose-derived mesenchymal stem cells; hUC-MSCs, human umbilical cord derived mesenchymal stem cells; CTD, connective tissue disorders; ILD, interstitial lung disease; ANCA, antineutrophil cytoplasmic antibodies; IPAF, idiopathic pneumonia with autoimmune features; 6MWT, 6-min walk test; CT, computed tomography.

Due to the physical properties of MSCs, exogenous engraftment of MSCs *in vivo* after infusion seems to be rare at the site of injury, thus hampering the therapeutic efficacy of MSCs. To avoid cell-related problems, novel applications of MSC-derived EVs has gained interest in tissue fibrosis treatment in recent years. EVs that are 30–5,000 nm in diameter include exosomes (30–100 nm), microvesicles/ectosomes (100–1,000 nm), and apoptotic bodies (100–5,000 nm). EVs can carry a variety of substances including mRNAs, microRNAs, enzymes, and other bioactive molecules (Abraham and Krasnodembskaya, 2020; Kanai et al., 2021). An EV of endocytosis origin is called an exosome, and an EV that forms from outward budding of the plasma membrane is called a microvesicle or microparticles (Gao and Raj, 2020). MSCs-derived EVs can be administered after isolation, characterization, and purification from the conditioned medium (CM) of cultured MSCs. However, there are limited studies on the effect of MSC-EVs on IPF. Mansouri and colleagues proposed that MSC-EVs can shift pro-inflammatory/classical and non-classical monocytes, as well as the alveolar macrophages, in the lung towards monocyte/macrophage profiles, which may reduce cell apoptosis, reverse PF, and improve the Ashcroft score (Mansouri et al., 2019). The effects of MSC-EVs on lung fibroblasts were investigated by Wan et al. (2020), and the results suggested that MSC-EVs can inhibit proliferation, migration, invasion, and differentiation of fibroblasts through overexpression of miR-29-3p in IPF.

The underlying mechanisms of PF, including telomere shortening and EMT, have also been considered research hotspots. The biological process of IPF has been described as an aberrant repair response to repeated alveolar epithelial injury in genetically susceptible aging individuals. Type II alveolar epithelial cells (AEC2) serve as the primary progenitor/stem cells for alveolar regeneration during tissue repair and homeostasis (Selman and Pardo, 2006; Barkauskas et al., 2013). In IPF, AEC2 display telomere shortening, epigenetic changes, and cellular senescence, which lead to impaired renewal capacity and dysfunction. Telomeres are the protective end caps of eukaryotic chromosomes and determine the proliferative lifespan of somatic cells, functioning as the protectors of cell replication (Celtikci et al., 2021). Genetic studies have confirmed that significant telomere shortening was found in 40% of familial IPF cases and 25% of sporadic IPF cases. Moreover, genetic alterations in telomere-related genes (e.g., TERT, TERC, TINF2, RTEL1, PARN, etc.) have been identified. Regulator of telomere length 1 (RTEL1), which is an essential helicase, has been implicated in sporadic IPF (Wang et al., 2020). Telomere shortening leads to the senescence of alveolar epithelial stem cells, and AEC2 apoptosis leads to a significant reduction in cell number, resulting in reduced alveolar regeneration (Zhang et al., 2021). In addition, senescent AEC2 secrete high levels of interleukins, interferons, tumor necrosis factors, colony-stimulating factors, growth factors, TGF-β, and chemotactic cytokines, which promote the differentiation of fibroblasts into myofibroblasts and ongoing tissue remodeling (Zhang et al., 2022). Therefore, telomere dysfunction and AEC2 cell senescence are the main drivers of IPF (Michalski and Schwartz, 2020). Telomerase gene therapy treatment in wild-type and telomerase-deficient mice has been demonstrated to prevent the development of pulmonary profibrotic lesions (Liu et al., 2019; Piñeiro-Hermida et al., 2020). EMT plays an important role in the development of IPF as well. EMT is a process in which fully differentiated epithelial cells gradually transform into mesenchymal cells and lose their original function after exposure to extracellular factors (Nieto et al., 2016), which is an important mechanism leading to the generation of myofibroblasts. During the EMT process, epithelial cells undergo significant changes in transcriptional regulation, cytoskeleton shape, adhesion, and ECM components (Sacks et al., 2018).

## Discussion

To the best of our knowledge, this is the first bibliometric study to review the research trend of MSCs in the field of PF. Our findings reveal the important discoveries, areas of research interest, and research frontiers of PF-related studies. We analyzed a total of 1,457 articles with 7,842 authors and found that during the past 2 decades, the literature involving MSCs and PF increased annually, especially after 2016. This indicates that the research on MSCs and PF is growing, and MSCs are likely to become a prominent research direction in the future treatment of PF.

Our bibliometic analysis showed that the United States (first) and China (second) were the top two countries conducting research on MSCs and PF. The top 10 institutions (60%) were from the United States, and only one institution was from China. In addition, we identified close cooperation between the top four countries (United States, China, Germany, and Italy), suggesting that there is comprehensive collaboration in the research field of MSCs and PF. Our analysis also demonstrates cooperation among several institutions. An extensive cooperation should be carried out to promote the development of MSCs research in the field of PF in China, which would facilitate a better collaboration with recognized Chinese institutes.

Most of the studies were published in *PLOS ONE*, and there were many papers published in journals with high impact factor, including the *American Journal of Respiratory and Critical Care Medicine* (IF = 30.528, Q1) and *Journal of Clinical Investigation* (IF = 19.456, Q1). Regarding co-cited academic journals, most studies were from journals with high impact factors (Q1 region). These journals have a significant international influence and could determine the future direction of MSCs and PF research.

Current studies that have investigated the role of MSCs in PF were mainly published in journals focused on the topics of molecular biology and immunology, and a small number was published in medical journals. This indicates that the application of MSCs remains largely at the level of bench research, and only several studies have entered translational stages. Therefore, the utilization of MSCs in the management of PF may be clinically available soon.

The most common keywords, including IPF, MSCs and systemic sclerosis, and others described previously represent the current research direction and research interests. Importantly, the efficacy and safety of MSCs for the treatment of PF have been confirmed in animal studies (Harrell et al., 2019; Yang et al., 2021; Zhao et al., 2021). Further cluster analysis of keywords showed that the role of MSCs in the management of PF may be explained by the following mechanisms: 1) Various growth factors, including keratinocyte growth factor, hepatocyte growth factor, epidermal growth factor, and angiogenic factors secreted by MSCs, lead to re-epithelialization and angiogenesis (Cahill et al., 2016; Lan et al., 2017; Li et al., 2017); 2) MSCs could express a series of receptors, intercellular contact molecules, and soluble factors [e.g., Toll-like receptors (Shirjang et al., 2017)] that regulate the adaptive and innate immune system by inhibiting the maturation of T-cells and dendritic cells, reducing the activation and proliferation of B cells, and inhibiting the cytotoxicity of natural killer cells (Ni et al., 2018; de Castro et al., 2019; He et al., 2020); 3) MSCs exert an immunosuppressive effect by increasing the level of interleukin (IL)-10 secreting regulatory B cells. In addition, MSCs could release a series of anti-inflammatory cytokines and chemokines, such as IL-1 receptor antagonist (Harrell et al., 2020), IL-4, IL-10, interferon (IFN)-γ, and prostaglandin (PGE)-2; 4) MSCs could reduce the proportion of cells with a profibrotic phenotype (Type 2 macrophages) to exert antifibrotic effects (Willis et al., 2018). Moreover, MSCs can reduce the content of collagen fibers and inhibit lung refactoring through adjusting the proportion of metalloproteinases/metal protease inhibitors, thus antagonizing fibrosis (Xu et al., 2017; Chu et al., 2019); 5) MSCs can interact with endothelial cells and epithelial cells, inhibit EMT, and promote angiogenesis and alveolar repair (Wei et al., 2021).

As reported in previous human studies, the injection of stem cells is safe in practice. Although the positive role of MSC therapy has been acknowledged in some literature, the efficacy of cell therapy varies (Campo et al., 2021) (Averyanov et al., 2020). In most studies, autologous or allogeneic bone marrow MSCs with different doses ranging from 1 to 200 × 10^6^ cells were administrated via different routes (Tzouvelekis et al., 2013; Glassberg et al., 2017; Averyanov et al., 2020; Campo et al., 2021). To date, most clinical studies remain in the early stage, and stem cell therapy still needs to be evaluated for its safety, feasibility, tolerability, and efficacy. It should be noted that limitations exist widely in these studies, including small sample size, lack of randomization, and/or placebo control. Furthermore, stem cell therapy requires optimization, including the type of stem cells, *in vitro* modifications, intervention protocol, route of administration, and dosage (Saleh et al., 2022). EVs and functional enhanced MSCs, such as those overexpressing CXCR4 (Zhang et al., 2019) and CXCR7 (Shao et al., 2019) or those with endocytosis of superparamagnetic iron oxide nanoparticles (Silva et al., 2018), have better therapeutic effects and may become a hot spot in the field of MSCs research in the future. Overall, current studies support that MSC therapy is safe for PF treatment and some patients could benefit from the therapy. However, since IPF is a disease with great heterogeneity, it is critical to identify subgroups that can benefit from stem cell therapy.

In this study, we systematically analyzed the literature related to the role of MSCs in the management of PF using a bibliometrics approach. Our analysis provides comprehensive insight for clinicians and scholars in the research field of MSCs and PF. Scientometrics provides a descriptive and quantitative analysis and yields more evidence on the status of the research field compared to the traditional narrative commentary. When interpreting our results, several limitations should be taken into consideration. For example, only one database (WoSCC) was searched, only literature published in English was included, and only literature published in the past 2 decades was included. These limits could lead to potential bias.

Overall, our findings provide insight into the status and research trends in the field of MSC and PF during the past 2 decades. Our findings could help guide future preclinical and clinical studies on the application of MSCs in the treatment of PF.

## Data Availability

The raw data supporting the conclusion of this article will be made available by the authors, without undue reservation.

## Abbreviations

6MWT, 6-min walk test; AD-MSCs, adipose-derived mesenchymal stem cells; ANCA, antineutrophil cytoplasmic antibodies; BM-MSCs, bone marrow-derived mesenchymal stem cells; CT, computed tomography; CTD, connective tissue disorders; DLco, diffusing capacity of the lung for carbon monoxide; FVC, functional vital capacity; hUC-MSCs, human umbilical cord derived mesenchymal stem cells; IFN, interferon; IL, interleukin; ILD, Interstitial Lung disease; IPAF, idiopathic pneumonia with autoimmune features; IPF, idiopathic pulmonary fibrosis; IV, intravenous; MSCs, mesenchymal stem cells; PARN, poly(A)-specific ribonuclease; PF, pulmonary fibrosis; PGE, prostaglandin; RTEL1, regulator of telomere length 1; TERC, telomerase RNA component; TERT, telomerase reverse transcriptase; TGF, tumor growth factor.
